# How to Plant Apple Trees to Reduce Replant Disease in Apple Orchard: A Study on the Phenolic Acid of the Replanted Apple Orchard

**DOI:** 10.1371/journal.pone.0167347

**Published:** 2016-12-01

**Authors:** Chengmiao Yin, Li Xiang, Gongshuai Wang, Yanfang Wang, Xiang Shen, Xuesen Chen, Zhiquan Mao

**Affiliations:** 1 State Key Laboratory of Crop Biology, College of Horticulture Science and Engineering, Shandong Agricultural University, Taian, Shandong, China; 2 College of Chemistry and Material Science, Shandong Agricultural University, Taian, Shandong, China; College of Agricultural Sciences, UNITED STATES

## Abstract

Apple replant disease (ARD) is an important problem in the production of apple. The phenolic acid is one of the causes of ARD. How phenolic acid affects the ARD was not well known. In this study, we analyzed the type, concentration and annual dynamic variation of phenolic acid in soil from three replanted apple orchards using an accelerated solvent extraction system with high performance liquid chromatography (ASE-HPLC). We found that the type and concentration of phenolic acid were significantly differed among different seasons, different sampling positions and different soil layers. Major types of phenolic acid in three replanted apple orchards were phlorizin, benzoic acid and vanillic aldehyde. The concentration of phenolic acid was highest in the soil of the previous tree holes and it was increased from the spring to autumn. Moreover, phenolic acid was primarily distributed in 30–60 cm soil layer in the autumn, while it was most abundant in 0–30 cm soil layer in the spring. Our results suggest that phlorizin, benzoic acid and vanillic aldehyde may be the key phenolic acid that brought about ARD in the replanted apple orchard.

## Introduction

When an apple orchard ages, orchard renewing and replanting are inevitable. The issues for apple replant disease (ARD) are the syndromes that occur when replanting in the same location, primarily manifesting as slow growth of apple plant shoots, weakened growth or even plant death, and that eventually shorten the life of the apple orchard [1–2]. ARD is a universal challenge in production, causing huge economic losses to farmers and severely constraining the sustainable development of fruit production [3]. Identifying ways to effectively mitigate or overcome ARD has become an important task in the sustainable development of apple production [4].

There are many complex pathogenic factors that cause ARD, and some of the possible factors are variable between the different regions or orchards of the same region [5]. Accumulated research results suggest that ARD is not attributed to only one factor, but rather to consortium of abiotic elements and biotic forces [6–7]. Biotic factors included nematodes, oomycetes bacteria, and fungi species [4, 5, 8, 9]. Abiotic factors such as soil structure, nutrition, and the release of allelochemicals through leaching, root exudation, volatilization, and/or decomposition of residues played roles in replant problems [10–11]. Studies have shown that allelochemicals enhanced superoxide radical (O_2_^·-^), H_2_O_2_ and MDA levels and increased membrane leakage in the plant tissues [12–13]. Phenolic acid is the secondary metabolites of apple roots and is important allelochemicals. Early studies found that phenolic acid including phloretin, phlorizin and amygdalin generated by apple roots can strongly inhibit the growth of apple seedlings [14]. Phenolic acid from root exudates and decomposed roots of previous crops to be one of the cause of ARD [6, 13, 15]. Yang et al. [16] found that ferulic acid, p-hydroxybenzoic acid and p-coumaric acid inhibited the accumulation of chlorophyll of rice leaves, and these phenolic acids harmed apple plants when accumulated to a certain concentration [17–18].

Here we analyzed and determined the type, concentration and dynamic variation of the phenolic acid in three replanted apple orchards using an accelerated solvent extraction system along with high performance liquid chromatography to understand the changes of the phenolic acid and to provide a theoretical basis for renewing the old apple orchard.

## Materials and Methods

### Ethics Statement

No specific permits were required for the three described field studies. All experiments were performed according to institutional guidelines of Shandong Agricultural University, China.

### Sample collection

Three 20-year-old orchards were chosen from Damozhuang village of Ciyao town in Ningyang county (E 35.77° N 116.8°), Xuanzhuang village of Daolang town in Daiyue district (E 36.18° N 117.0°) in Taian city, Shandong province, China and Jincheng town of Laizhou city (E 37.18° N 119.93°) in Yantai, Shandong province, China. The old trees were removed, and 2-year-old Fuji/*Malus* apple seedlings were replanted to rebuild the orchards. Samples were collected from the previous tree holes (tree holes), sites between the previous rows (inter-rows) and sites between the previous trees (inter-trees) at 2 soil layers: 0–30 cm and 30–60 cm, and times at the spring, summer and autumn in the year of 2012 and 2013. The soils were sieved, placed in zip-top bags, stored in the dark and brought back to the laboratory for analysis. Geochemical and physical characteristics of soil samples were determined (S1 Table). Concentration of phenolic acid was determined in three orchards soil without planting trees in January 2012 (S2 Table). There were three replicates for each sample.

### Accelerated solvent extraction (ASE)

The soil samples were dried and about 100 g soil was used for analysis. The appropriate amount of diatomaceous earth was added to the sample and mixed well in a beaker. A cellulose membrane was placed at the bottom of a 100 ml extraction cell, the well-mixed sample was placed in the extraction cell, and the extraction was conducted according to the following ASE conditions. The absolute ethanol was used as the extraction solvent under a temperature of 120°C, pressure of 10.3 Mpa and a static extraction time of 5 min in 2 cycles, with a purge volume of 60% and purge time of 90 s. The methanol was used as the extraction solvent to extract the same sample under the same conditions. After the extraction was completed, the liquids collected from the two extractions were mixed and concentrated under reduced pressure at 34°C to nearly dry. Then, 1 ml of methanol was added to re-dissolve the sample, and the solution was passed through a 0.22 μm organic membrane filter. The sample was ready for HPLC analysis [19].

### Chromatographic conditions

The conditions of measurement were as follows. The chromatographic column is acclaim 120 C_18_ (3 μm, 150 mm × 3 mm); column temperature is 30°C; mobile phase is A: acetonitrile and B: water (the pH was adjusted to 2.6 using acetic acid); flow rate is 0.5 ml min^-1^; injection method is autosampler; injection volume is 5 μl; detection wavelength is 280 nm [19].

### Statistical analysis

The data are presented as the means±one standard deviation (SD) of three replicates. The statistical analyses, such as analysis of variance (ANOVA), were performed using the SPSS software (version 19.0, SPSS Inc., Chicago, USA). Duncan’s multiple range test (DMRT) was applied to compare the significant differences between treatments.

## Results

### Types and concentrations of phenolic acid in the soil of three replanted orchards in the spring

Types of phenolic acid in three replanted orchards soil were different in the spring (Table 1). Ciyao replanted orchard was composed of 10 phenolic acids, including p-hydroxy benzoic acid, syringic acid, vanillic aldehyde, coumaric acid, ferulic acid, benzoic acid, salicylic acid, phlorizin, cinnamic acid and phloretin. Daolang replanted orchard was composed of 12 phenolic acids, including p-hydroxy benzoic acid, syringic acid, vanillic aldehyde, coumaric acid, ferulic acid, benzoic acid, salicylic acid, phlorizin, cinnamic acid, phloretin, caffeic acid and chlorogenic acid. Jincheng replanted orchard was composed of 14 phenolic acids, including p-hydroxy benzoic acid, syringic acid, vanillic aldehyde, coumaric acid, ferulic acid, benzoic acid, salicylic acid, phlorizin, cinnamic acid, phloretin, caffeic acid, coumarin, catechin and phloroglucinol.

**Table 1:** Types and concentration of phenolic acid in the soil of Ciyao, Daolang and Jincheng replanted orchards in the spring.

Types and concentration of phenolic acid (mg/kg)	Ciyao inter-rows		Ciyao tree hole		Ciyao inter-trees		Daolang inter-rows		Daolang tree hole		Daolang inter-trees		Jincheng inter-rows		Jincheng tree hole		Jincheng inter-trees	
soil layer	0–30	30–60	0–30	30–60	0–30	30–60	0–30	30–60	0–30	30–60	0–30	30–60	0–30	30–60	0–30	30–60	0–30	30–60
P-hydroxy benzoic acid	0.53d	0.29c	—	—	0.33cd	0.07e	0.25c	—	0.35e	0.29def	0.15bc	0.07d	0.35f	0.27d	0.27de	0.08d	0.22d	—
Syringic acid	0.07g	0.02g	0.14ef	0.04c	0.05e	0.03e	0.06f	—	0.42e	0.25f	0.07c	0.20a	0.16gh	0.38d	0.12ef	—	0.08ef	—
Vanillic aldehyde	0.88c	0.44b	0.98b	0.33bc	0.76b	0.41b	0.53b	0.09b	1.43b	0.99b	0.44a	0.19ab	1.27c	0.60c	0.69c	0.22d	0.75c	0.21b
Coumaric acid	0.36e	0.16de	0.38cde	0.08c	0.34c	0.13d	0.22c	0.02b	0.62cd	0.25f	0.24b	—	0.40f	0.08e	0.12ef	—	0.10e	—
Ferulic acid	0.13fg	0.05fg	0.24def	0.06c	0.12e	0.03e	0.09ef	0.01b	0.43e	0.26ef	0.07c	0.02e	0.15gh		0.07f	—	0.07ef	—
Benzoic acid	1.50a	1.35a	1.90a	0.40b	1.14a	0.64a	0.63a	—	2.76a	1.56a	0.57a	—	2.27b	0.52c	1.14b	1.59b	—	0.59b
Salicylic acid	0.35e	0.13def	0.50cd	0.22bc	0.28cd	0.28c	0.18d	—	0.70c	0.37d	0.15bc	0.04de	2.41a	3.85a	3.73a	3.18a	3.11a	4.10a
Phlorizin	0.22f	0.20cd	0.56c	5.54a	0.17de	0.14d	0.13e	1.36a	0.59d	0.87c	0.13bc	—	0.09gh	—	—	1.36c	—	—
Cinnamic acid	0.12fg	0.07efg	0.10f	0.04c	0.17de	0.04e	0.08ef	0.01b	0.19f	0.07g	0.08bc	0.01e	0.63e	—	—	—	—	—
Phloretin	1.14b	0.05fg	0.44cd	0.31bc	—	—	—	0.08b	0.26f	0.35d	—	0.15c	0.81d	0.91b	0.30d	—	1.70b	—
Caffeic acid	—	—	—	—	—	—	—	—	—	0.02g	—	—	0.18g	0.09e	0.06f	—	—	—
Coumarin	—	—	—	—	—	—	—	—	—	—	—	—	0.18g	0.06e	0.08f	—	0.05f	—
Catechin	—	—	—	—	—	—	—	—	—	—	—	—	0.12gh	—	—	—	0.09ef	—
Phloroglucinol	—	—	—	—	—	—	—	—	—	—	—	—	0.07h	0.06e	0.07f	—	—	—
Chlorogenic acid	—	—	—	—	—	—	—	—	—	0.33de	—	0.16bc	—	—	—	—	—	—

Data are the means of three replicates (±SD), different letters indicate significant differences at P < 0.05.

Types of phenolic acid were different among different sampling sites in the same orchard (Table 1). In Ciyao and Jincheng replanted orchards, the types of phenolic acid were more in the inter-rows than in the tree holes or inter-trees, while in the Daolang replanted orchard, most types of phenolic acid were in the tree holes. The types of phenolic acid were more in the 0–30 cm soil layer than in the 30–60 cm soil layer in the replanted orchards of Daolang and Jincheng, while they were same in the 0–30 cm and 30–60 cm layers in replanted orchard of Ciyao.

The concentration of phenolic acid was also different among three replanted orchards in different soil layers and different sampling sites in the spring (Table 1). In Ciyao replanted orchard, phlorizin, benzoic acid, vanillic aldehyde, phloretin and p-hydroxy benzoic acid were most abundant. In Daolang replanted orchard, phlorizin, benzoic acid, vanillic aldehyde, salicylic acid and coumaric acid were most abundant. In Jincheng replanted orchard, phlorizin, benzoic acid, vanillic aldehyde, phloretin and salicylic acid were most abundant. The concentrations of phenolic acid were higher in the 0–30 cm soil layer than in the 30–60 cm soil layer of all three replanted orchards in the spring.

### Types and concentrations of phenolic acid in the soil of three replanted orchards in the summer

Types of phenolic acid in three replanted orchards were also different in the summer (Table 2). There was no change in the types of phenolic acid in Ciyao, while four phenolic acids were decreased in Jincheng and two phenolic acids were decreased in Daolang compared with the spring. In Ciyao and Jincheng replanted orchards, the types of phenolic acid were less in the inter-rows than in the tree holes or inter-trees, while in the Daolang replanted orchard, most types of phenolic acid were in the inter-rows. The types of phenolic acid were same in the 0–30 cm and 30–60 cm layers of all three replanted orchards.

**Table 2:** Types and concentration of phenolic acid in the soil of Ciyao, Daolang and Jincheng replanted orchards in the summer.

Types and concentration of phenolic acid (mg/kg)	Ciyao inter-rows		Ciyao tree hole		Ciyao inter-trees		Daolang inter-rows		Daolang tree hole		Daolang inter-trees		Jincheng inter-rows		Jincheng tree hole		Jincheng inter-trees	
soil layer	0–30	30–60	0–30	30–60	0–30	30–60	0–30	30–60	0–30	30–60	0–30	30–60	0–30	30–60	0–30	30–60	0–30	30–60
P-hydroxy benzoic acid	—	—	—	—	0.36b	0.15b	0.51ab	0.05c	—	—	—	—	0.21c	0.57c	1.30c	0.56d	0.63b	0.36d
Syringic acid	0.06e	0.02e	0.14f	0.27f	0.03e	0.46a	0.08b	0.02c	0.39ef	0.22f	—	—	0.09de	0.01g	0.55e	0.55d	0.23d	0.26e
Vanillic aldehyde	0.66b	0.41b	1.18c	1.98b	0.39b	0.43a	0.33ab	0.25bc	1.64b	1.46b	0.64b	0.36a	0.34b	0.81b	1.58b	1.40b	0.41c	0.81b
Coumaric acid	0.35cd	0.13d	0.53d	1.24c	0.12cd	0.11b	0.37ab	0.04c	1.11c	0.57d	0.16de	0.07c	0.12cde	0.40d	0.95d	0.61cd	0.16de	0.26e
Ferulic acid	0.16de	0.06e	0.32e	0.58de	0.06de	0.03b	0.19ab	0.01c	0.51def	0.34e	0.07e	0.06c	0.09de	0.23e	0.66e	0.65c	0.22d	0.09fg
Benzoic acid	1.56a	1.23a	2.11a	4.01a	0.99a	0.63a	0.53a	0.44b	3.55a	3.30a	0.97a	0.05c	1.18a	2.09a	3.88a	3.75a	2.38a	2.93a
Salicylic acid	0.41c	0.24c	0.63d	1.30c	0.18c	0.08b	0.40ab	0.02c	0.85cd	0.69d	0.30c	0.12b	0.16cd	0.16ef	0.56e	0.44e	0.39c	0.49c
Phlorizin	0.31cd	0.15d	1.34b	0.76d	0.13c	0.18b	0.17ab	0.29bc	0.68de	1.06c	0.24cd	0.05c	0.02e	0.11f	0.15g	0.34f	0.03e	0.06g
Cinnamic acid	0.12e	0.05e	0.28ef	0.41ef	0.06de	0.03b	0.13ab	0.04c	0.19f	0.18f	0.16de	0.06c	0.03e	0.47d	0.63e	0.47e	0.05e	0.15f
Phloretin	0.07e	—	0.24ef	0.30f	0.05e	—	0.08b	0.76a	0.14f	0.03g	—	—	—	0.12f	0.34f	0.35f	0.13de	0.09fg

Data are the means of three replicates (±SD), different letters indicate significant differences at P < 0.05.

The concentration of phenolic acid was also different among three replanted orchards in different soil layers and different sampling sites in the summer (Table 2). In Ciyao replanted orchard, phlorizin, benzoic acid, vanillic aldehyde, salicylic acid and coumaric acid were most abundant. In Daolang replanted orchard, phlorizin, benzoic acid, vanillic aldehyde, salicylic acid and coumaric acid were most abundant. In Jincheng replanted orchard, phlorizin, benzoic acid, vanillic aldehyde, p-hydroxy benzoic acid and coumaric acid were most abundant.

### Types and concentrations of phenolic acid in the soil of three replanted orchards in the autumn

Types of phenolic acid in three replanted orchards were also different in the autumn (Table 3). There was no change in the types of phenolic acid in Daolang, while four were increased in Jincheng and one phenolic acid was increased in Ciyao compared with the summer. Most types of phenolic acid were in the tree holes of all three replanted orchards. The types of phenolic acid were more in the 0–30 cm than 30–60 cm layers in three replanted orchards in the autumn.

**Table 3:** Types and concentration of phenolic acid in the soil of Ciyao, Daolang and Jincheng replanted orchards in the autumn.

Types and concentration of phenolic acid (mg/kg)	Ciyao inter-rows		Ciyao tree hole		Ciyao inter-trees		Daolang inter-rows		Daolang tree hole		Daolang inter-trees		Jincheng inter-rows		Jincheng tree hole		Jincheng inter-trees	
soil layer	0–30	30–60	0–30	30–60	0–30	30–60	0–30	30–60	0–30	30–60	0–30	30–60	0–30	30–60	0–30	30–60	0–30	30–60
P-hydroxy benzoic acid	0.22ef	0.07d	—	0.22c	—	0.13b	—	—	—	—	—	—	0.38c	0.49c	0.40e	0.57c	0.26d	0.13d
Syringic acid	0.17ef	—	0.46e	0.11c	0.20d	0.04b	0.05e	—	0.22f	0.76g	0.04f	—	0.13d	0.10f	0.19f	0.10f	0.09d	0.14d
Vanillic aldehyde	1.01b	0.16c	1.52b	0.70c	1.25b	0.43b	0.58b	0.33b	1.30b	2.12bc	0.87a	0.16b	1.20b	0.96b	0.92d	0.92b	0.99c	0.35c
Coumaric acid	0.34de	0.04d	0.94c	0.44c	0.66c	0.17b	0.18d	0.07d	0.44e	0.78g	0.21d	0.04c	0.09d	0.16e	0.26ef	0.16ef	0.13d	0.05d
Ferulic acid	0.28ef	0.02d	0.49de	0.19c	0.27d	0.06b	0.07e	0.03d	0.38e	1.10fg	0.11e	—	0.15d	0.16e	0.19f	0.11f	0.12d	—
Benzoic acid	1.89a	0.51b	4.03a	3.14b	2.51a	—	1.52a	0.45a	2.39a	1.59de	0.82b	0.74a	—	0.23d	3.81b	—	3.16b	6.67a
Salicylic acid	0.54cd	0.03d	1.00c	0.50c	0.62c	0.05b	0.45c	0.15c	0.74c	4.55a	0.36c	0.07c	3.05a	7.29a	4.39a	7.14a	4.03a	5.87b
Phlorizin	0.69c	0.67a	0.85cd	15.94a	0.35d	7.64a	0.23d	0.16c	0.62d	2.35b	0.20d	0.03c	—	—	1.67c	0.34d	—	—
Cinnamic acid	0.15ef	0.02d	0.24ef	0.07c	0.17de	0.05b	0.19d	0.04d	0.20f	1.36ef	0.23d	0.05c	—	—	0.15f	0.19e	—	—
Phloretin	0.07f	0.05d	0.51de	3.60b	0.02e	0.43b	—	0.04d	—	1.87cd	—	—	—	—	4.44a	0.93b	0.07d	—
Caffeic acid	—	—	0.03f	—	0.01e	—	—	—	—	—	—	—	—	—	0.27ef	—	—	—
Coumarin	—	—	—	—	—	—	—	—	—	—	—	—	0.12d	0.17e	0.15f	—	—	—
Phloroglucinol	—	—	—	—	—	—	—	—	—	—	—	—	—	—	0.14f	—	0.08d	0.13d

Data are the means of three replicates (±SD), different letters indicate significant differences at P < 0.05.

The concentration of phenolic acid was also different among three replanted orchards in different soil layers and different sampling sites in the autumn (Table 3). Phlorizin, benzoic acid, vanillic aldehyde, phloretin and salicylic acid were most abundant in the soil of all three replanted orchards. The concentrations of phenolic acid were higher in the 30–60 cm soil layer than in the 0–30 cm soil layer of all three replanted orchards.

### Changes of total phenolic acid concentration of the soil sampled at different sampling sites among different seasons and different years

**Fig 1:**
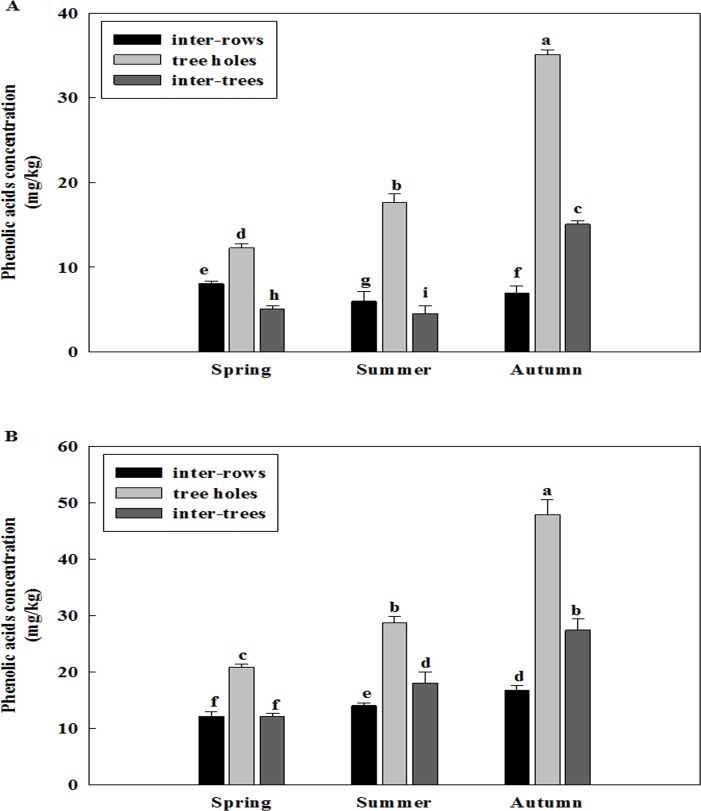
Changes of total phenolic acid concentration of the soil of Ciyao replanted orchard at different sampling sites among different seasons. A: Samples collected in the 2012, B: Samples collected in the 2013. Data are the means of three replicates (±SD), different letters indicate significant differences at P < 0.05.

**Fig 2:**
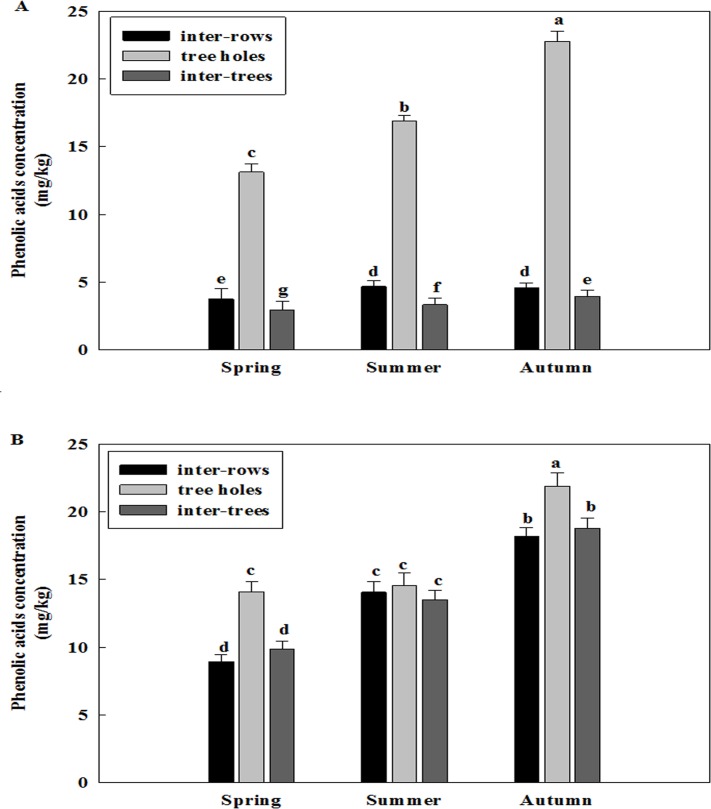
Changes of total phenolic acid concentration of the soil of Daolang replanted orchard at different sampling sites among different seasons. A: Samples collected in the 2012, B: Samples collected in the 2013. Data are the means of three replicates (±SD), different letters indicate significant differences at P < 0.05.

**Fig 3:**
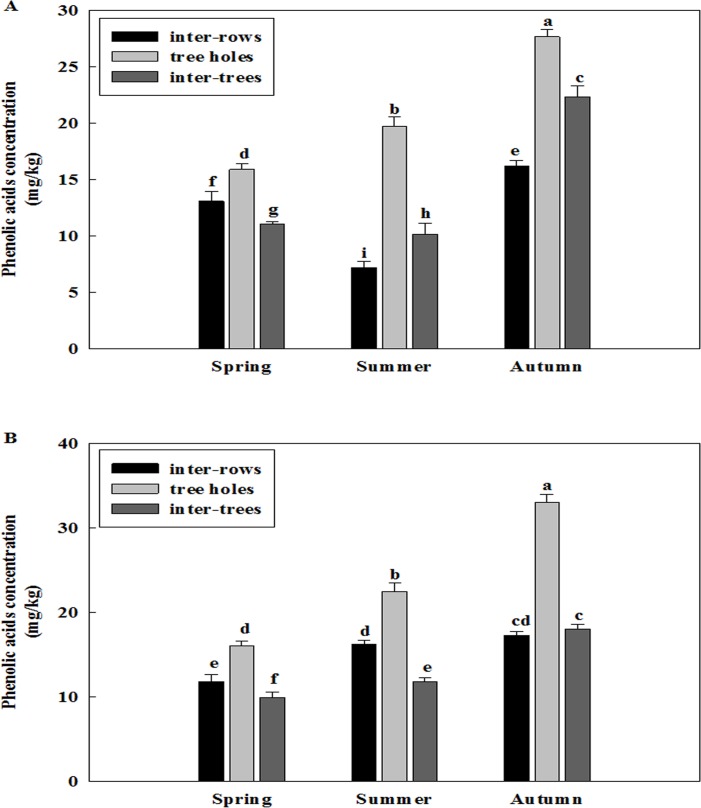
Changes of total phenolic acid concentration of the soil of Jincheng replanted orchard at different sampling sites among different seasons. A: Samples collected in the 2012, B: Samples collected in the 2013. Data are the means of three replicates (±SD), different letters indicate significant differences at P < 0.05.

Total phenolic acid concentration was different in three replanted orchards among the seasons of spring, summer and autumn (Figs 1A–3A). The concentration of phenolic acid was highest in the soil of the previous tree holes and it was increased from the spring to autumn of all three replanted orchards. Overall, the concentration of total phenolic acid was higher in the autumn than in the spring and summer.

Total phenolic acid concentration was also different in three replanted orchards among the years of 2012 and 2013 (Figs 1A–3A and 1B–3B). The concentration of phenolic acid was highest in the soil of the previous tree holes and it was increased from the 2012 to 2013 of all three replanted orchards. Overall, the concentration of total phenolic acid was higher in the 2013 than in the 2012.

### Total phenolic acid concentration changes in different soil layers among different seasons and different years

**Fig 4:**
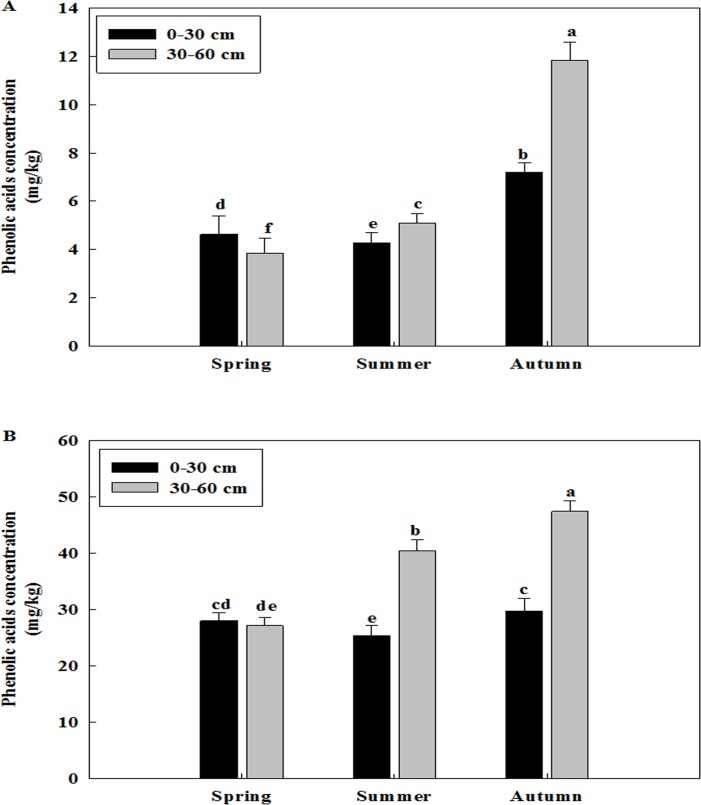
Changes of total phenolic acid concentration of Ciyao replanted orchard in different soil layers among different seasons. A: Samples collected in the 2012, B: Samples collected in the 2013. Data are the means of three replicates (±SD), different letters indicate significant differences at P < 0.05.

**Fig 5:**
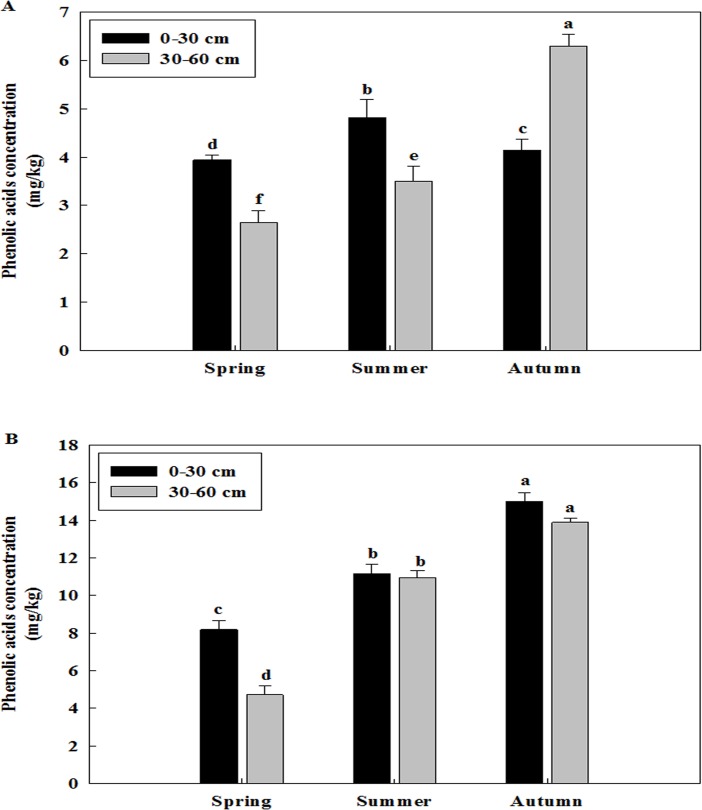
Changes of total phenolic acid concentration of Daolang replanted orchard in different soil layers among different seasons. A: Samples collected in the 2012, B: Samples collected in the 2013. Data are the means of three replicates (±SD), different letters indicate significant differences at P < 0.05.

**Fig 6:**
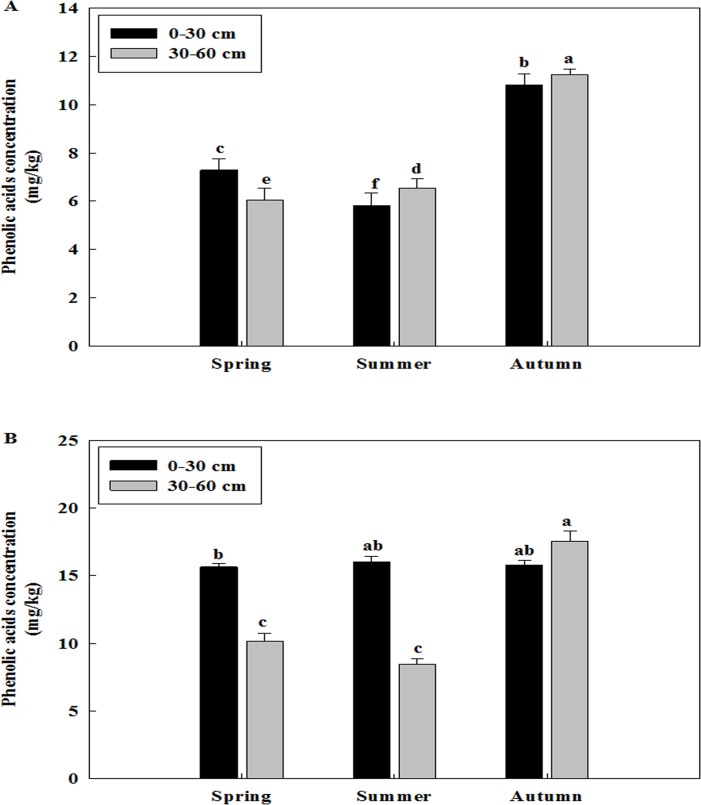
Changes of total phenolic acid concentration of Jincheng replanted orchard in different soil layers among different seasons. A: Samples collected in the 2012, B: Samples collected in the 2013. Data are the means of three replicates (±SD), different letters indicate significant differences at P < 0.05.

Total phenolic acid concentration was different in two soil layers of three replanted orchards among the seasons of spring, summer and autumn (Figs 4A–6A). The concentration of total phenolic acid of two soil layers was higher in the autumn than in the spring and summer of all three replanted orchards. The concentration of phenolic acid was primarily distributed in 30–60 cm soil layer in the autumn, while it was most abundant in 0–30 cm soil layer in the spring of all three replanted orchards.

Total phenolic acid concentration was also different in three replanted orchards among the years of 2012 and 2013 (Figs 4A–6A and 4B–6B). The concentration of phenolic acid was highest in the 30–60 cm soil layer in the autumn and it was increased from the 2012 to 2013 of all three replanted orchards. Overall, the concentration of total phenolic acid was higher in the 2013 than in the 2012.

### Determination of biomass of the replanted apple trees in three replanted apple orchards

Plant height, dry perimeter, the average branch length and fresh and dry weight of the replanted apple trees significantly differed among inter-rows, inter-trees, tree holes in three replanted apple orchards (Table 4). Compared with different sampling sites, the growth of replanted trees in Daolang inter-rows was best, and plant height of the replanted apple trees in Daolang inter-rows were increased 1.32 times, 1.10 times than those in Ciyao and Jincheng inter-rows, respectively. Compared with different sampling sites, plant height, dry perimeter, numbers of branches of the replanted trees in tree holes were lower than inter-rows, and plant height of the replanted apple trees in Ciyao tree holes were decreased 0.84 times, 0.78 times than those in Ciyao inter-rows, Ciyao inter-trees, respectively.

**Table 4:** Biomass of the replanted trees was determined in three apple replanted orchards in the 2013.

Sample location	Treatment	Height (cm)	Dry perimeter (cm)	Average branch length(cm)	Ground fresh weight(g)	Underground fresh weight(g)	Ground dry weight (g)	Underground dry weight (g)
Ciyao	Inter-rows	163.2d	8.8c	77.3de	528c	321c	293e	194d
	Tree holes	137.1e	7.3d	61.5e	331e	240e	193f	129f
	Inter-trees	175.5d	8.8c	71.6de	466d	302d	334d	173e
Daolang	Inter-rows	214.8a	11.6a	121.5b	1804a	566a	971a	381a
	Tree holes	178.5bcd	8.7c	66.4de	552c	300d	558c	246c
	Inter-trees	195.2b	10.2b	100.3c	699b	358b	749b	341b
Jincheng	Inter-rows	194.8bc	12.1a	142.1a	—	—	—	—
	Tree holes	177.5cd	11.5a	73.3de	—	—	—	—
	Inter-trees	175.1d	10.6b	80.1d	—	—	—	—

Data are the means of three replicates (±SD), different letters indicate significant differences at P < 0.05.

## Discussion and Conclusion

Plants released phenolic acid into soils by leaching from the aboveground part, excreting from the roots and via decay of residual crops [11, 20, 21]. Studies showed that certain concentrations of the phenolic acid could make the cucumber seedling mitochondria, plastids, nuclear membrane and endoplasmic reticulum membrane damaged to different extent, including the changes in membrane structure and function [22–23]. Vanillic acid, coumaric acid, caffeic acid and syringic acid significantly inhibited the growth of primary and secondary roots of green beans at a certain concentration. Hiradate et al. [17] found that a large amount of phenolic acid accumulated in soil could make the plant antioxidant system destroyed under replanted condition. In our study, we analyzed and determined the type, concentration and dynamic variation of the phenolic acid in three replanted apple orchards. Ourresults showed that types of phenolic acid in three replanted orchards were different among the seasons of spring, summer and autumn, suggest that different types of phenolic acids secreted by plants in different seasons [24].

Phenolic acid could directly affect the status of soil nutrients and plant growth through microbial and pest activity, leading to the occurrence of ARD [25–27]. Baerson et al. [20] showed that phenolic acid secreted by apple roots play an important role in the ARD, and these phenolic acids could harm the plants at a certain concentration [17–18]. Gao et al. [13] found that cinnamic acid could inhibit the basal respiration rate of seedling roots at a certain concentration in *Malus hupehensis* Rehd. Our results showed that the concentration of phenolic acid was highest in the 30–60 cm soil layer in the autumn and it was increased from the 2012 to 2013 of all three replanted orchards. This result was consistent with the results of Kuiters [28], so the accumulation of phenolic acids in soil was an important cause of ARD [25].

The severity of ARD in the replanted orchards was related to the position of the replanted young trees and the old tree holes [29]. Our results showed that the concentration of phlorizin, benzoic acid, vanillic aldehyde were most abundant in the soil of the tree holes in three replanted orchards and it was increased from the spring to autumn. Phlorizin, benzoic acid, vanillic aldehyde may be the key phenolic acids that caused ARD in three replanted orchards. Avoiding the tree holes when replanting apples could reduce the incidence of ARD to a certain extent.

## Supporting Information

S1 Table Geochemical and physical characteristics of soil samples in three replanted orchards. (DOCX)

S2 Table Concentration of phenolic acids in three orchards soil without planting trees in January 2012. (DOCX)
