# Supplementary material for: How to Plant Apple Trees to Reduce Replant Disease in Apple Orchard: A Study on the Phenolic Acid of the Replanted Apple Orchard
Source: PLoS One. 2016 Dec 1;11(12):e0167347. doi: 10.1371/journal.pone.0167347 (PMC5132267; doi:10.1371/journal.pone.0167347)
Supplement: S1 Table — (DOCX) [file pone.0167347.s001.docx]

**S1 Table Geochemical and physical characteristics of soil samples.**

|  | NO-N | NH-N | P | K | Organic matter |
| --- | --- | --- | --- | --- | --- |
| Treatments | mg/kg | mg/kg | mg/kg | mg/kg | g/kg |
| Ciyao inter-rows 0-30 | 24.970±3.800ijkl | 2.480±0.190ab | 24.530±0.580g | 98.520±2.410g | 7.680±0.360d |
| Ciyao inter-rows 30-60 | 21.320±1.840jkl | 2.160±0.430abcd | 19.670±0.640i | 55.450±1.180m | 6.770±0.140f |
| Ciyao tree holes 0-30 | 28.190±1.340ghijk | 1.870±0.160abcd | 24.920±0.320g | 104.970±0.860f | 7.410±0.230de |
| Ciyao tree hole 30-60 | 19.890±3.060kl | 1.530±0.110cd | 16.890±0.880k | 55.740±0.430m | 6.280±0.190g |
| Ciyao inter-trees 0-30 | 32.490±1.530efghi | 2.010±0.440abcd | 27.570±0.550f | 107.090±1.890f | 7.390±0.430de |
| Ciyao inter-trees 30-60 | 16.430±1.870l | 1.440±0.190d | 17.920±0.170j | 63.950±0.440l | 6.380±0.190g |
| Daolang inter-rows 0-30 | 42.850±0.760bcd | 1.360±0.200d | 29.790±0.890d | 119.380±0.400e | 8.790±0.230ab |
| Daolang tree holes 0-30 | 15.550±1.030l | 1.700±0.450bcd | 18.690±0.350j | 83.430±0.340j | 7.000±0.220ef |
| Daolang tree holes 0-30 | 51.310±1.900ab | 1.590±0.160bcd | 44.320±0.200b | 122.770±0.410d | 8.290±0.170c |
| Daolang tree holes 30-60 | 34.980±1.860defgh | 2.700±0.210a | 21.860±0.110h | 80.300±0.400k | 7.160±0.430ef |
| Daolang inter-trees 0-30 | 30.560±1.750fghij | 1.860±0.100abcd | 29.100±0.250d | 163.130±1.120b | 8.120±0.100c |
| Daolang inter-trees 30-60 | 15.910±2.730l | 1.760±0.090bcd | 22.440±0.350h | 83.770±0.680j | 7.170±0.440ef |
| Jincheng inter-rows 0-30 | 40.620±1.100cde | 1.590±0.140bcd | 39.710±0.280c | 123.410±1.440d | 8.270±0.180c |
| Jincheng inter-rows30-60 | 38.990±1.550def | 2.460±0.350abc | 24.530±0.150g | 97.570±0.560gh | 7.290±0.290de |
| Jincheng tree holes 0-30 | 37.140±1.180defg | 2.190±0.220abcd | 28.960±0.110d | 141.190±1.440c | 9.140±0.190a |
| Jincheng tree holes 30-60 | 27.180±1.060hijkl | 1.930±0.110abcd | 22.500±0.230h | 95.430±0.900h | 8.090±0.120c |
| Jincheng inter-trees 0-30 | 52.020±2.220a | 1.970±0.180abcd | 50.160±0.410a | 208.310±1.430a | 8.460±0.230bc |
| Jincheng inter-trees30-60 | 49.030±1.400abc | 1.400±0.120d | 24.920±0.140g | 88.110±1.040i | 7.370±0.110de |

Data are the means of three replicates (±SD), different letters indicate significant differences at P < 0.05.
