# Supplementary material for: How to Plant Apple Trees to Reduce Replant Disease in Apple Orchard: A Study on the Phenolic Acid of the Replanted Apple Orchard
Source: PLoS One. 2016 Dec 1;11(12):e0167347. doi: 10.1371/journal.pone.0167347 (PMC5132267; doi:10.1371/journal.pone.0167347)
Supplement: S2 Table — (DOCX) [file pone.0167347.s002.docx]

**S2 Table Concentration of phenolic acids in three orchards soil without planting trees in January 2012.**

| Phenolic acid concentration (mg/kg) | Ciyao inter-rows | Ciyao tree hole | Ciyao inter-trees | Daolang inter-rows | Daolang tree hole | Daolang inter-trees | Jincheng inter-rows | Jincheng tree hole | Jincheng inter-trees |
| --- | --- | --- | --- | --- | --- | --- | --- | --- | --- |
| 0-30 cm | 4.80c | 4.74c | 2.86e | 1.59f | 6.80a | 1.32f | 6.88a | 5.77b | 3.92d |
| 30-60 cm | 2.26d | 6.52a | 1.27e | 0.98e | 4.69c | 0.18f | 5.35b | 4.57c | 4.40c |

Data are the means of three replicates (±SD), different letters indicate significant differences at P < 0.05.
